# Maternal Death Surveillance and Response: Looking Backward, Going Forward

**DOI:** 10.9745/GHSP-D-23-00099

**Published:** 2023-04-28

**Authors:** Matthews Mathai

**Affiliations:** a Associate Editor, Global Health: Science and Practice Journal; Independent consultant, St. John’s, NL, Canada.

See related article by Tura et al.

Understanding why a woman died during or immediately after pregnancy and childbirth is a crucial first step toward preventing other women from dying in the same way. Besides understanding the medical cause of death, it is also important to know the woman’s personal story and the other nonmedical factors that may have contributed to her death.

In 2012, the World Health Organization (WHO) and partners launched maternal death surveillance and response (MDSR) as an approach to end preventable maternal mortality. MDSR follows a continuous cycle of notification, review, analysis, and response. Many low- and middle-income countries (LMICs) moved quickly to adopt MDSR in their national policies and practices.^1^ By 2016, 85% of LMICs reportedly had a national policy to review all maternal deaths. However, only 46% of LMICs reportedly had national maternal death review committees that met at least biannually, thus highlighting the need for countries to follow through on their policy commitments and “complete the loop” in the surveillance-response cycle.^2^

Ethiopia introduced national MDSR in 2013. It was among the first sub-Saharan countries to capture maternal deaths at the facility and community levels. The decision to make maternal mortality a weekly reportable condition within public health emergency management in 2014 led to its integration with the existing disease surveillance system in the country.^3^ However, the effective implementation of MDSR in Ethiopia has faced many challenges. While strong political support and leadership facilitated its introduction,^3^ reported barriers to implementation have included politicization of the reporting and review process, defensive attitudes, blame-shifting, poor attendance, and fear of legal repercussions.^4^^,^^5^

## ADAPTING OBSTETRIC SURVEILLANCE SYSTEMS FOR LMIC SETTINGS

In this issue of GHSP, Tura and colleagues^6^ report on a hospital-based obstetric surveillance system in eastern Ethiopia. Adapted for the Ethiopian context from obstetric surveillance systems used in high-income countries (HICs), the system collects information from 13 participating health care facilities in the region on selected maternal morbidities (hemorrhage, eclampsia, uterine rupture, sepsis, and severe anemia) and maternal deaths. Ten maternal deaths and 904 women with the morbidities of interest were reported among 17,317 live births in these facilities in the first 6 months. Staff were also trained to conduct confidential enquiries into maternal deaths (CEMD).

While this externally funded hospital-based surveillance project has worked well in the short term in 1 region, it is unclear how this approach will, in the longer term, contribute to improved maternal survival across Ethiopia. Considerable financial support would be needed to run another surveillance system in parallel to an existing national system. Long-term sustainability will require buy-in from the government and closer integration with national surveillance systems.

Tura et al. report that the existing national MDSR system reportedly captures less than 10% of maternal deaths. Underreporting of maternal deaths, albeit at lesser levels, occurs even in HICs.^7^ However, ensuring corrective actions (the “response”) are taken after reviewing even a small proportion of deaths could contribute to improvement to maternal health and survival.

The authors’ statement that “…while the MDSR is a facility-based review of maternal death by a multidisciplinary committee to identify the cause of the death and design an appropriate response for preventing similar deaths at that facility in the future, CEMD focuses on more general recommendations that extend beyond the review of a single case at a specific facility” misinterprets WHO guidance on MDSR.^8^ Well-functioning MDSR systems also include maternal deaths outside health facilities and corrective actions to recommend that go beyond the health care facility. Unlike HICs, where most births take place in hospitals and maternal deaths are rare, half of births in Ethiopia are still at home.^9^ The CEMD process, as reported by Tura et al., focuses only on maternal deaths in hospitals.

## ESTABLISHING MDSR TAKES TIME

MDSR systems take time to establish and will encounter challenges in early implementation. WHO guidance on MDSR recommends a phased approach to implementation.^8^ The importance to “think big but to start small and grow gradually” cannot be overemphasized. Many LMICs have aspired to implement CEMD at the national level without first establishing good confidential (“no name”) and nonpunitive (“no blame”) audit practices at the facility and community levels. The first maternal death reviews in the United Kingdom were initiated by health care workers in Rochdale in northwestern England who were concerned with having the highest rate of maternal mortality in the country.^10^ This local practice was adopted in other jurisdictions over the following 2 decades leading to the establishment of the first national CEMD in 1952. Obstetric surveillance of maternal morbidities was established several decades later, only after maternal deaths had become uncommon.

To develop functional MDSR, think big but start small and grow gradually.

Successful MDSR implementation can be expected to lead to improved quality of care and reduced maternal mortality. As noted by Willcox and colleagues^11^ “the key context to enable effective death surveillance and response was a blame-free learning environment with good leadership.” The politicization of maternal death reviews and the fear of punitive actions and litigations, as reported from Ethiopia,^4^^,^^5^ can be demotivating and lead to “a vicious cycle of under-reporting, inaccurate data, and inadequate review and recommendations, which lead to demotivation and disengagement.”^11^ Lessons from this project and from global experiences on the facilitators and barriers to successful implementation should inform efforts to make the Ethiopian national MDSR system more effective.
